# XNAT imaging platform for BioMedBridges and CTMM TraIT

**DOI:** 10.1186/2043-9113-5-S1-S18

**Published:** 2015-05-22

**Authors:** Stefan Klein, Erwin Vast, Johan van Soest, Andre Dekker, Marcel Koek, Wiro Niessen

**Affiliations:** 1 Biomedical Imaging Group Rotterdam, Departments of Medical Informatics & Radiology, Erasmus MC, 3000CA Rotterdam, the Netherlands; 2 Maastricht UMC+, MAASTRO Clinic, Maastricht, the Netherlands; 3 Imaging Physics, Applied Sciences, Delft University of Technology, the Netherlands

## Characterisation

Service, imaging, open source, archive, image sharing, clinical trials.

## Description

**Figure 1:**
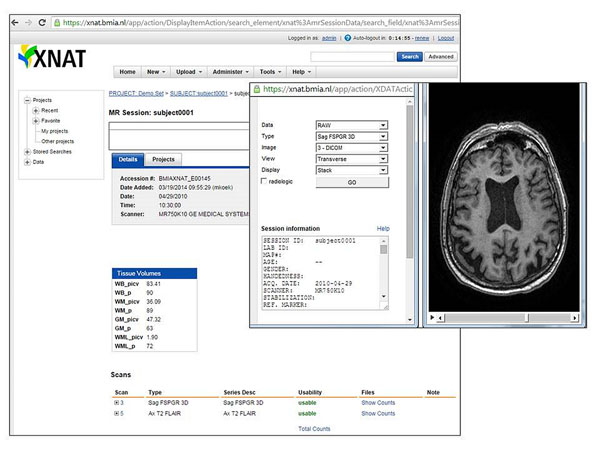
XNAT user interface. Example project with magnetic resonance imaging (MRI) data of the brain.

The XNAT imaging platform is a web service for storing, organizing, and sharing medical imaging data. It is based on the open source eXtensible Neuroimaging Archive Toolkit (XNAT, http://www.xnat.org) [1]. The platform is suitable for both single-center and multi-center clinical studies with imaging data (Figure 1). Medical imaging data (in DICOM format) and image-derived data (often in non-DICOM format) are supported. Downloading and uploading is possible via the web interface, via the DICOM protocol, and via a RESTful application programming interface. Read and write access rights can be controlled per project and per user. In the context of the European BioMedBridges project and the Dutch CTMM TraIT project, two XNAT instances have been put in production. In the CTMM TraIT instance, which is especially supports multi-center clinical imaging studies, submission of DICOM imaging data is performed via the RSNA Clinical Trial Processor (CTP) to enforce proper anonymisation. In order to simplify and automate the installation of XNAT, we developed scripts based on the “Puppet” configuration software. These scripts have been made publicly available via the XNAT marketplace (http://marketplace.xnat.org).

## Status of development

A recent stable release of the XNAT software is used. Currently (since November 2014), we are running version 1.6.2.1. The XNAT instances are in use by several research projects. The configuration scripts are in active development, and the current release is version 0.2 alpha.

## Users

Researchers using medical imaging data.

## Links

Documentation and links to both XNAT instances and Puppet installation scripts [http://xnat.bigr.nl]; documentation of RSNA CTP [http://mircwiki.rsna.org/index.php?title=CTP_Articles].
